# High-quality carrots in relation to the superior phloem parenchyma cells and proportion of xylem vessel in coordinated water-fertilizer management

**DOI:** 10.3389/fpls.2025.1590774

**Published:** 2025-06-13

**Authors:** Ying Zhao, Yanchen Gong, Yun Li, Hua Xin, Ruiping Chi, Yanling Chen

**Affiliations:** ^1^ College of Resources and Environment Science, Qingdao Agricultural University, Qingdao, Shandong, China; ^2^ Agricultural Technology Extension Service Center, Laixi Agriculture and Rural Bureau, Qingdao, China

**Keywords:** carrot, flavor quality, nutritional quality, parenchyma cell, xylem vessel

## Abstract

**Background:**

The coordinated management of water and fertilizer is essential for improving vegetable yield and quality. However, its role in connecting quality with the anatomical structure of the phloem and xylem in carrots remains unclear.

**Methods:**

This study involved a two-year field trial with four different water and fertilizer treatments: farmer practices (FP), an optimized water and fertilizer system (OPT), 30% organic substitution with compressed peanut shells (PS), and 30% organic substitution with Pleurotus ostreatus residue (M) combined with water and fertilizer optimization.

**Results:**

Compared with the FP treatment, the OPT, PS, and M treatments increased both yield and quality. Flavor quality increased by 17.51%, 13.04%, and 15.05%, and nutritional quality increased by 11.04%, 8.12%, and 17.35% in the upper, middle, and lower segments, respectively, in the OPT treatment. In contrast, the organic substitution treatments (average of PS and M) resulted in even greater improvements, with flavor quality increasing by 32.50%, 18.21%, and 38.07%, and nutritional quality increasing by 10.28%, 4.69%, and 25.41%, respectively. In the phloem, flavor and nutritional quality increased by 9.59% and 13.50%, respectively, in the OPT treatment and by 12.35% and 17.69%, respectively, in the organic substitution treatment. In the xylem, flavor and nutritional quality increased by 1.64% and 19.09%, respectively, in the OPT treatment, whereas in the organic substitution treatments, flavor quality increased by 16.89%, and nutritional quality increased by 1.94%. Compared with those in the FP treatment, the phloem parenchyma cell area (Pca) and the proportion of xylem vessels to secondary xylem (Pxv) in the upper segment were 9.17% and 88.40% greater in the OPT treatment, respectively, and 18.44% and 116.22% greater in the organic substitution treatment, respectively. The parameters characterizing Pca and Pxv in the upper segment, along with Pca in the lower segment, were positively correlated with flavor and nutritional quality, whereas the xylem vessel area (Xva) and diameter (Xvd) were negatively correlated.

**Conclusion:**

In conclusion, the coordinated management of water, organic, and inorganic fertilizers improves flavor and nutritional quality across the upper, middle, and lower segments, as well as in the phloem and xylem. The superior phloem parenchyma cell area and xylem vessel proportion in the upper segment may serve as physiological traits in breeding carrots for quality improvement.

## 1 Introduction

Carrots are well-known vegetable crops worldwide and are prized for their abundance of nutrients such as carotene, amino acids, and vitamins (Zhang et al., 2020). China accounts for almost 40% of the global carrot planting area (FAO, 2022). Nevertheless, in recent years, farmers have been using excessive water and fertilizers more frequently to increase yields, leading to issues related to crop quality and the environment (Rietra et al., 2017). The optimization of water and fertilizer management is crucial for decreasing input costs while improving both yield and quality (Gan et al., 2023).

Carotenoid levels in the aboveground and root sections are higher with fractional nitrogen (N) fertilizer treatments than with one-third N treatments (Colombari et al., 2018). Compared with conventional methods, organic management offers greater commerciality, reduced waste, and increased vitamin C content in carrots (Olle and Williams, 2021). The evaluation of high-quality carrots is based on their appearance, flavor, and nutritional quality (Carrillo-López and Yahia, 2019). The shape of carrot taproots is primarily determined by the balance of length, elongation, and thickness, leading to various root types ranging from circular to conical or cylindrical (Kjellenberg et al., 2016). Cylindrical roots are regarded as a standard for appearance quality (Thompson, 1969). Flavor quality is determined by the balance between sweetness and acidity (Kader, 2008).

Carrot quality and the distribution of the main primary and secondary metabolites vary significantly across genotypes and radially distributed tissues (Aubert et al., 2022). In two carrot genotypes, the malic acid content is greater in the xylem than in the phloem, whereas α-carotene, β-carotene, and carotenoids are more abundant in the phloem (Aubert et al., 2022; Perrin et al., 2017). The levels of metabolites, sucrose, and carotenoids decrease from the top to the bottom of the tissue. Among the three carrot varieties, carotenes are most concentrated in the secondary phloem (Kim et al., 2010). However, the understanding of carrot quality across vertical segments and radial distribution under optimal water-fertilizer management is limited, which is crucial for the cultivation of high-yield and high-quality carrots.

The formation and expansion of carrot roots involve structural changes, material accumulation, and gene regulation (Khadr et al., 2020). The vascular cambium divides the root into two parts: the secondary phloem (cortex) and the secondary xylem (core) (Perrin et al., 2017). The xylem transports water, whereas the phloem carries amino acids and sucrose (Zhao et al., 2005). The phloem expands significantly during growth, especially with increasing parenchyma cell number and volume (Suojala, 2000). Its metabolic function surpasses that of the xylem, resulting in the accumulation of more nutrients (Aubry et al., 2019). Sugars are mainly stored in parenchyma vacuoles (Nilsson, 1987), and carotenoids are synthesized in chloroplasts (Perrin et al., 2017). However, the relationships between the phloem and xylem structure and quality in carrots remain unclear.

Previous studies have shown that optimizing water and fertilizer management can greatly improve the overall quality of carrots in long-term experiments (Tang et al., 2024). This study delves deeper into the underlying physiological mechanisms by examining the following hypotheses: a) optimized water-fertilizer management enhances flavor and nutritional quality across different vertical segments of carrots, and b) a superior phloem and xylem structure in the horizontal radial distribution promotes the accumulation of soluble solids, sugars, and carotene, contributing to excellent flavor and nutritional quality under coordinated water–fertilizer management.

## 2 Materials and methods

### 2.1 Field experiments

Field experiments were conducted in 2022 and 2023 as part of a long-term study on water and fertilizer management that was initiated in 2020 at the Science and Technology Backyard of LaiXi in Houtun village, Qingdao city, Shandong Province (36°N; 120°E). The experimental site has a temperate continental monsoon climate, with annual average temperatures of 13°C in 2022 and 14°C in 2023. The total rainfall during the two growing seasons was 715.5 mm in 2022 and 445.8 mm in 2023. The soil used in the experiment was mortar black soil, with the following properties at a depth of -20 cm: alkali-hydrolysable N, 175.7 mg·kg^-^¹; Olsen P, 27.7 mg·kg^-^¹; available K, 117.6 mg·kg^-^¹; pH, 6.80; and organic matter, 12.3 g·kg^-^¹.

*Pleurotus ostreatus* is abundant in nutrients, including protein, amino acids, vitamins, and various other substances that can be converted into organic fertilizer (Huang et al., 2023). Peanut shells are high in fiber, vitamins, flavonoids, and mineral nutrients such as calcium (Camargo et al., 2017). They can also serve as seedling substrate material postfermentation (Sun et al., 2003). Since local farmers cultivate peanuts and *Pleurotus ostreatus*, there is an abundance of residue available for utilization. The water and fertilizer treatments included (1) farmer practices (FP), where water and fertilizers are applied following traditional methods in Laixi; (2) an optimized water and fertilizer system(OPT), which uses real-time soil water or nutrient data and field capacity, employing sensors to fulfill carrot nutrient needs; (3) 30% organic substitution with compressed peanut shells + OPT (PS); and (4) 30% organic substitution with *Pleurotus ostreatus* residue + OPT (M). In the PS and M treatments, 30% of the N fertilizer was substituted with compressed peanut shells or *Pleurotus ostreatus* residue, maintaining total water and fertilizer levels similar to those in the OPT treatment. In 2022 and 2023, FP application rates were established at 323 kg N ha^-^¹, 225 kg P_2_O_5_ ha^-^¹, and 285 kg K_2_O ha^-^¹ on the basis of local surveys (**Table 1**). The OPT rates were 252 and 236 kg N ha^-^¹, 54 and 59 kg P_2_O_5_ ha^-^¹, and 240 and 300 kg K_2_O ha^-^¹, as determined by carrot growth stages, respectively. The PS rates were 252 and 236 kg N ha^-^¹, 63 and 74 kg P_2_O_5_ ha^-^¹, and 240 and 300 kg K_2_O ha^-^¹, respectively. The application rates of the M treatment were 252 and 236 kg N ha^-^¹, 78 and 88 kg P_2_O_5_ ha^-^¹, and 240 and 300 kg K_2_O ha^-^¹, respectively. Urea (N 46%) was used as the N fertilizer, diammonium phosphate (N 18%, P_2_O_5_ 46%) was used as the P source, and potassium sulfate (K_2_O 50%) was used for K. The organic fertilizer consisted of an agricultural microbial agent (N 4%). The agricultural microbial agents were applied in FP and OPT at a rate of 1200 kg ha^-1^. In 2022 and 2023, the PS treatment was applied at rates of 4740 kg·ha^-1^ and 4440 kg·ha^-1^ peanut shell compressed particles, whereas the M treatment was applied at rates of 4995 kg·ha^-1^ and 4680 kg·ha^-1^ mushroom residue. The fertilizer is applied in liquid form by dissolving the powder in water. This experiment utilized elemental fertilizer to achieve the necessary N, P, and K content ratios for optimal carrot growth.

**Table 1:** Total input of nutrient and water in each treatment in 2022 autumn and 2023 spring.

Year/Treatments		2022 Autumn				Water and fertilizers reduction (%)	2023 Spring				Water and fertilizers reduction (%)
		FP	OPT	PS	M		FP	OPT	PS	M	
N	(kg ha^-1^)	323	252	252	252	-21.9	323	236	236	236	-26.8
P_2_O_5_	(kg ha^-1^)	225	54	63	78	-71.1	225	59	74	88	-67.4
K_2_O	(kg ha^-1^)	285	240	240	240	-15.8	285	300	300	300	5.3
Water	(m^3^ ha^-1^)	924	862	894	875	-5.1	1835	1587	1605	1587	-13.2

Water and fertilizers reduction = (the average of water and fertilizers input amount in the OPT, PS, M treatments – the water and fertilizers input amount in the FP treatment)/the water and fertilizers input amount in the FP treatment.

In terms of water management, the FP treatment utilized customary irrigation volumes, whereas the remaining treatments were modified according to the real-time soil moisture and field capacity data provided by sensors. The total irrigation volumes were 924 and 1835 m³·ha^-^¹ for the FP treatment, 862 and 1587 m³·ha^-^¹ for the OPT treatment, 894 and 1605 m³·ha^-^¹ for the PS treatment, and 875 and 1587 m³·ha^-^¹ for the M treatment, in 2022 and 2023. Details of the nutrient and water inputs at each stage can be found in **Supplementary Table S1**.

In this experiment, four large plots, each measuring 220.5 m^2^, were established. Each large plot was divided into four repetitions. The carrot varieties Zhenjiuhong in 2022 and Hongta313 in 2023 were selected on the basis of their climate adaptability in spring and autumn. Planting was conducted on raised ridges, with each ridge supporting two rows, resulting in a total of 7 ridges and 14 rows. The ridges had a spacing of 67–70 cm, height of 15 cm, top width of 28 cm, and spacing of 5–6 cm. Carrot seeds were sown at a depth of 1.5–2 cm on August 24, 2022, and January 9, 2023, and were harvested on December 10, 2022, and May 20, 2023, respectively.

### 2.2 Plant sampling and measurement

#### 2.2.1 Yield and plant sampling

**Figure 1:**
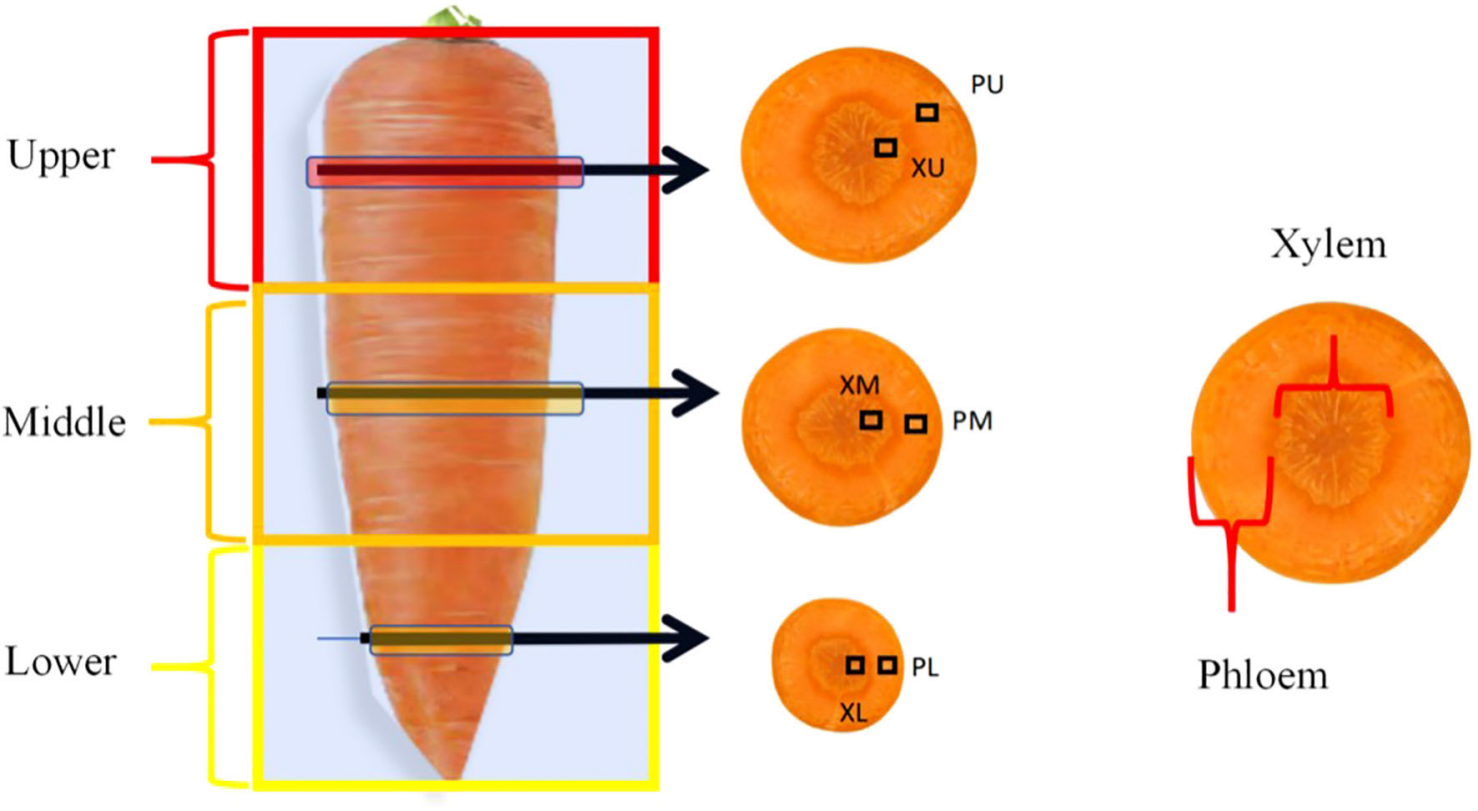
Sampling schematic diagram of phloem and xylem in the upper, middle and lower segments at equal proportion in carrot. PU, Phloem in the upper segment, XU, Xylem in the upper segment; PM, Phloem in the middle segment, XM, Xylem in the middle segment; PL, Phloem in the lower segment; XL, Xylem in the lower segment.

At maturity, the central four rows (5 meters in length and 1.4 meters in width) in each plot were harvested. Carrots were extracted from the soil, counted, and weighed, and the yield per hectare was calculated on the basis of harvest density. Furthermore, fifteen uniform and representative carrot roots were collected and washed from each plot. One-third of these roots were utilized to assess appearance quality and then dried, after which the total N, total phosphorus (P), and total potassium (K) contents were measured. The total N content was determined using the Kjeldahl method. The total P content was analyzed using the molybdenum antimony colorimetric method. The total K content was measured via flame photometry (Bao, 2000). The remaining roots were divided as follows: five roots were segmented equally into upper, middle, and lower parts to analyze flavor and nutritional quality postjuicing with household appliances, while the remaining five roots were separated into phloem and xylem tissues using a peeler and knife (**Figure 1**). These tissues were preserved in formaldehyde-acetic acid–alcohol (FAA) fixative for observation of anatomical structure and evaluation of flavor and nutritional quality. Segmenting carrots into upper, middle, and lower parts for analysis aims to provide a more precise assessment of their nutrient composition and quality attributes, as well as to identify varietal differences. This process is crucial for carrot variety selection, food processing, and nutritional assessment.

#### 2.2.2 Quality measurement

Appearance Quality: The color index (Ci) was determined using an SC-10 colorimeter (Guangdong Sanenshi Technology Limited Company, China). Red-to-green tones were denoted as +a to –a, yellow-to-blue tones were represented as +b to –b, and brightness was denoted as L. Ci was calculated with the formula Ci = 1000a*/(Lb) (Passafiume et al., 2023). The compaction degree (Cd) was calculated as the ratio of the weight of a single root to the product of the root length and middle diameter. The root–tail convergence index (Rtci) was determined by the ratio of the bottom 0.5 cm to the top 0.5 cm. The pulp heart rate (Phr) was calculated as the ratio of the xylem diameter to the entire pulp diameter using a Vernier caliper.

Flavor Quality: The assessment of flavor quality, particularly the influence of sugars on taste, included the evaluation of total soluble solids (TSS) and the total soluble solids-to-titratable acid (TA) ratio (Beckles, 2012; Stevens et al., 1979). The TSS was determined using a handheld TD-32 sugar meter (Shanghai Lichen Bangxi Instrument Technology Limited Company, China). TA was measured through titration with sodium hydroxide. The TSS-to-TA ratio was computed to evaluate the equilibrium between soluble solids and organic acids (Baldwin et al., 1998).

Nutritional quality: Vitamin C content was determined by 2% oxalic acid extraction and titration with 2,6-dichloroindophenol (Ranganna, 1977). The carotene content was quantified using the acetone extraction method (Costache et al., 2012). Soluble protein levels were assessed using the Coomassie Brilliant Blue method (Sedmak and Grossberg, 1977), whereas free amino acids were quantified through ninhydrin colorimetry (Moore, 1968).

#### 2.2.3 Anatomical structure of the phloem and xylem

The carrot cross-sectional structure was analyzed using the paraffin sectioning method (Wang et al., 2016). Slices (5 mm thick) were taken from the longitudinal middle of the upper, middle, and lower segments of each sample and fixed. Half of the radial middle of the phloem and xylem in each slice was observed (**Figure 1**). The distribution, density, diameter, and area of phloem parenchyma cells (Pc) and xylem vessels (Ve) were quantified. The samples were dehydrated with a gradient of ethyl alcohol and xylene, embedded in paraffin, and sectioned to 8 µm using a Leica RM2126RT (Leica, Germany) (Yuan et al., 2023). The sections, which were stained with saffron O solid green, were visualized under an Olympus CX33 microscope (Olympus Corporation, Japan) and processed with MShot Image Analysis System software (Guangzhou Microshot Technology Limited Company, China), and cell measurements were conducted using ImageJ (National Institutes of Health). Nine visual fields per replicate were utilized to determine the Pc and vessel diameter, area, and secondary xylem proportion.

### 2.3 Statistical analysis

Data across water and fertilizer treatments and years were pooled and analyzed with a one-way ANOVA in the DPS data processing system. Differences were evaluated using the least significant difference test at a significance level of 0.05. Since the quality improvements were similar in the PS and M treatments, these improvements were averaged and considered organic alternatives in the analysis. Figures were generated in Origin 2024. Redundancy analysis (RDA) was performed in Canoco 5 (Ter Braak and Smilauer, 2002) to examine correlations between nutrient concentration, anatomical structure, flavor, and nutritional quality.

## 3 Results

### 3.1 Yield, nutrient concentration and quality of whole fleshy roots

**Figure 2:**
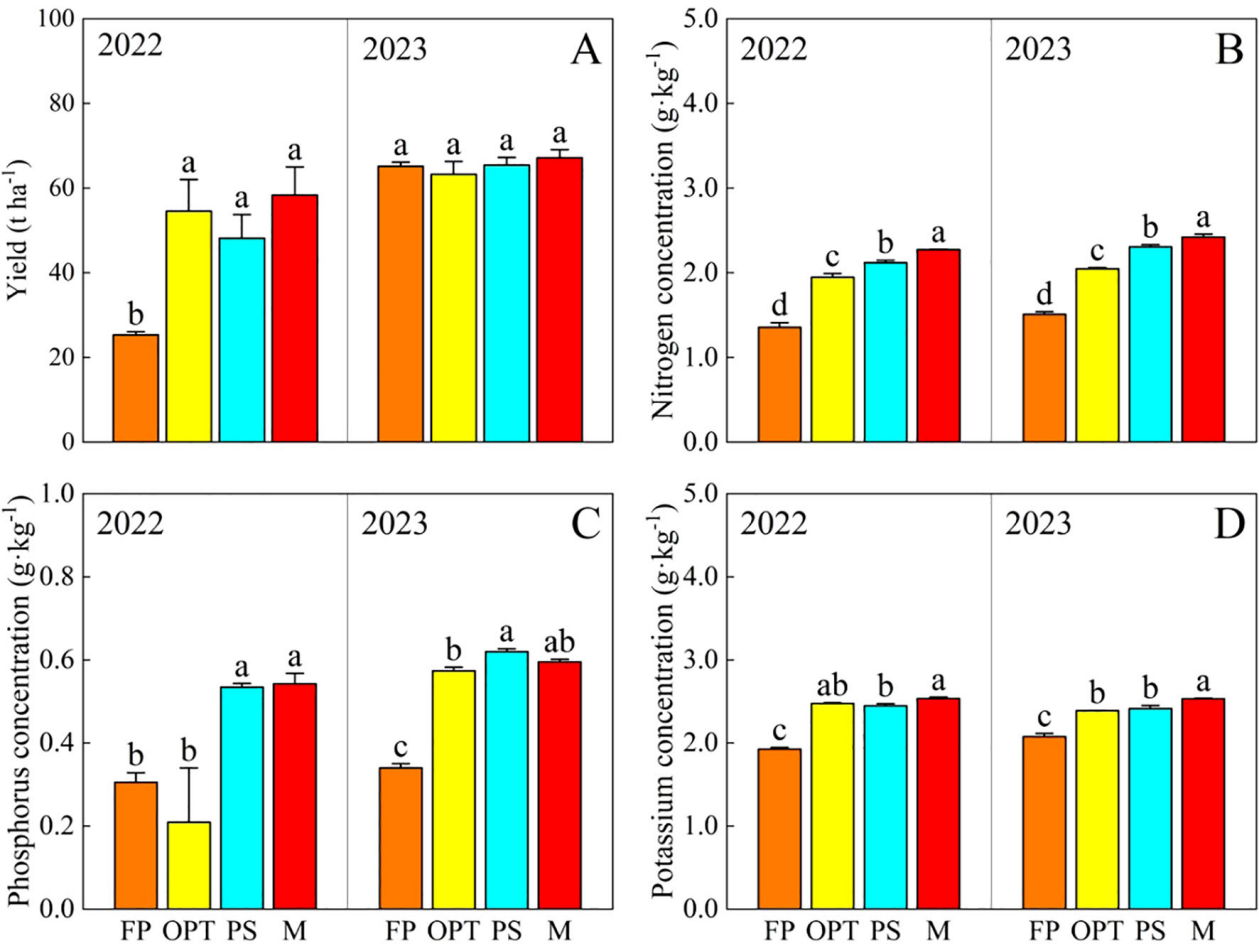
Effects of water and fertilizers management on yield and total nitrogen, total phosphorus, total potassium concentration in carrot. **(A–D)** represent yield, total nitrogen, total phosphorus, total potassium concentration in carrot, respectively. Different letters indicate significant differences among different water and fertilizers managements (P < 0.05). Values shown are means (n = 3 replicates).

**Figure 3:**
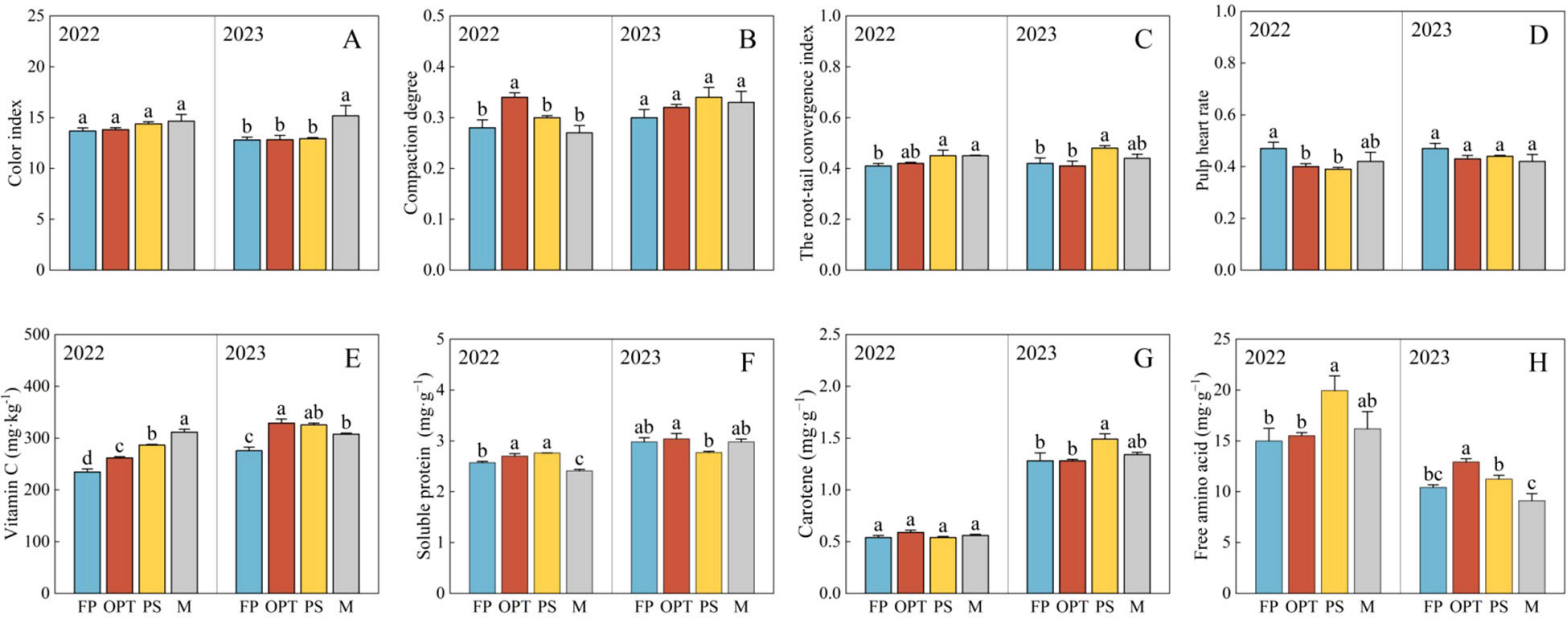
Effects of water and fertilizers management on appearance and nutritional quality in carrot. Different letters indicate significant differences among different water and fertilizers managements (P < 0.05). Values shown are means (n = 3 replicates). **(A–D)** represent color index, compaction degree, the root-tail convergence index, pulp heart rate of FP, OPT, PS and M treatments, respectively. **(E–H)** represent vitamin C, soluble protein, carotene, free amino acid of FP, OPT, PS and M treatments, respectively.

Compared with that in the FP treatment, the yield significantly increased in the OPT, PS, and M treatments in 2022 but not in 2023. There was no significant difference in yield between OPT and PS or M over the two years (**Figure 2A**). The total nitrogen (N) and total potassium (K) concentrations significantly increased in the OPT, PS, and M treatments in both years (**Figures 2B, D**). The total phosphorus (P) concentration significantly increased in the PS and M treatments in 2022, and it also significantly increased in the OPT, PS, and M treatments in 2023 (**Figure 2C**). Compared with that in the FP treatment, the Ci significantly increased in the M treatment but remained unchanged in the OPT and PS treatments in 2023 (**Figure 3A**). The Cd concentration significantly increased in the OPT treatment but remained unchanged in the PS and M treatments in 2022 (**Figure 3B**). The Rtci increased in the PS and M treatments in 2022, increased only in the PS treatment in 2023, and remained unchanged in the OPT treatment over the two-year period (**Figure 3C**). The Phr significantly decreased in the OPT and PS treatments but not in the M treatment in 2022 (**Figure 3D**). There was no significant difference in Ci in 2022 or in Cd or Phr in 2023 among the OPT and PS or M treatments (**Figures 3A-D**).

In terms of nutritional quality, compared with that in the FP treatment, the vitamin C concentration significantly increased in the OPT, PS, and M treatments in both years (**Figure 3E**). The soluble protein content significantly increased in the OPT and PS treatments but decreased in the M treatment in 2022 (**Figure 3F**). The carotene content significantly increased in the PS treatment but remained unchanged in the OPT and M treatments in 2023 (**Figure 3G**). The Free amino acid content significantly increased in the PS treatment but remained unchanged in the OPT and M treatments in 2022. In 2023, free amino acid content significantly increased in the OPT treatment but remained unchanged in the PS and M treatments (**Figure 3H**). No significant differences were found in the carotene content in 2022 or soluble protein content in 2023 between the OPT and PS or M treatments compared to FP treatment (**Figures 3F, G**).

In terms of flavor quality, compared with that in the FP treatment, the TSS content significantly increased in the PS treatment but remained unchanged in the OPT and M treatments in 2022. The TA significantly decreased in the OPT, PS, and M treatments in both years. The TSS-to-TA ratio significantly increased in the OPT, PS, and M treatments in both years. The soluble sugar content significantly increased in the PS and M treatments but remained unchanged in the OPT treatment in both years. Glucose levels significantly decreased in the OPT treatment group in 2023 but remained unchanged in the PS and M treatment groups. Sucrose levels significantly increased in the OPT and PS treatments but remained unchanged in the M treatment in 2022. In 2023, sucrose levels significantly increased in all the OPT, PS and M treatments. No significant differences were found in glucose levels in 2022 or in TSS levels in 2023 among the OPT and PS or M treatments (**Table 2**).

**Table 2:** Effects of water and fertilizers management on flavor quality in carrot.

Treatments/index		Total soluble solid (%)	Titratable acid (%)	TSS-to-titratable acid ratio	Soluble sugar (mg g^−1^ DW)	Glucose (mg g^−1^ DW)	Sucrose (mg g^−1^ DW)
2022	FP	7.86 ± 0.10b	0.84 ± 0.06a	10.65 ± 0.31b	403.82 ± 19.73b	149.99 ± 33.49a	154.52 ± 24.51b
	OPT	7.81 ± 0.13b	0.72 ± 0.02b	13.13 ± 0.73a	450.60 ± 17.29ab	121.97 ± 34.57a	209.71 ± 43.63a
	PS	8.31 ± 0.22a	0.64 ± 0.02bc	15.02 ± 1.21a	505.24 ± 58.82a	123.96 ± 53.92a	220.71 ± 13.81a
	M	7.96 ± 0.08b	0.57 ± 0.07c	15.02 ± 2.13a	503.27 ± 16.44a	127.99 ± 22.96a	191.72 ± 20.64ab
2023	FP	11.22 ± 0.34ab	1.52 ± 0.03a	7.43 ± 0.36d	536.08 ± 32.32b	195.88 ± 33.25a	137.05 ± 17.97b
	OPT	11.22 ± 0.12ab	1.15 ± 0.04b	9.82 ± 0.41c	595.61 ± 17.71b	117.24 ± 16.32b	228.27 ± 28.88a
	PS	11.53 ± 0.09a	0.95 ± 0.04c	12.41 ± 0.48a	679.92 ± 38.84a	243.84 ± 29.32a	238.30 ± 11.21a
	M	11.18 ± 0.07b	0.98 ± 0.04c	11.55 ± 0.40b	683.19 ± 34.53a	249.28 ± 31.41a	205.83 ± 4.83a

Different letters indicate significant differences among different water and fertilizers managements (P < 0.05). Values shown are means (n = 3 replicates).

### 3.2 Flavor and nutritional quality in the upper, middle, and lower segments

**Figure 4:**
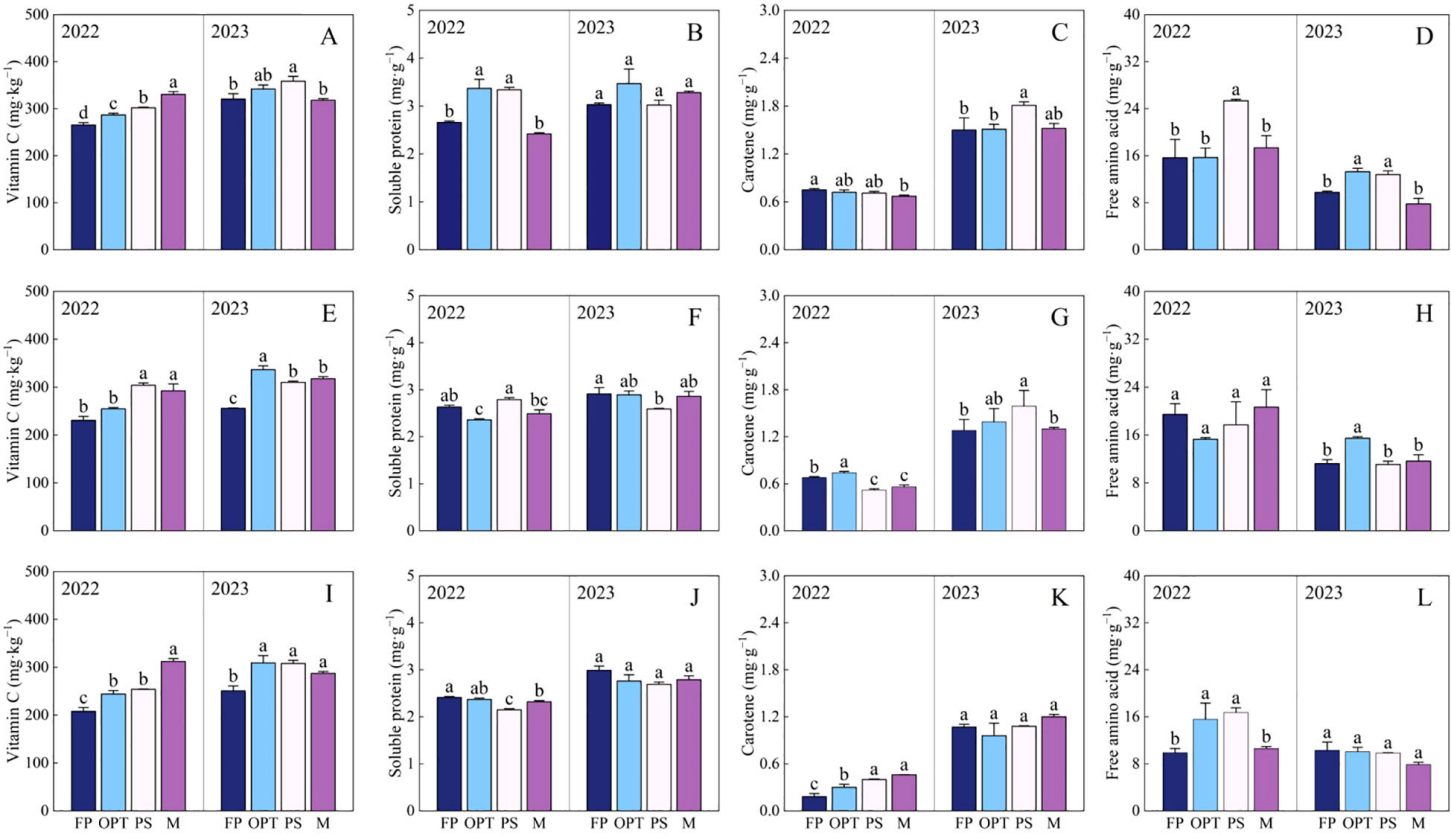
Effects of water and fertilizers management on nutritional quality in the upper, middle and lower segments in carrot. Different letters indicate significant differences among different water and fertilizers managements (P < 0.05). Values shown are means (n = 3 replicates). **(A–D)** represent nutritional quality in the upper of FP, OPT, PS, and M treatments, respectively. **(E–H)** represent nutritional quality in the middle of FP, OPT, PS, and M treatments of FP, OPT, PS, and M treatments, respectively. **(I–L)** represent nutritional quality in the lower of FP, OPT, PS, and M treatments.

Significant differences in flavor and nutritional quality were observed among the FP, OPT, PS, and M treatments (**Table 3**; **Figure 4**). In terms of flavor quality in the upper carrot segment, compared with that in the FP treatment, the TSS significantly increased in the PS treatment but remained unchanged in the OPT and M treatments in both years (**Table 3**). The TA significantly decreased in the OPT, PS, and M treatments in both years. The TSS-to-TA ratio significantly increased in the OPT and PS treatments but remained unchanged in the M treatment in 2022. However, in 2023, the TSS-to-TA ratio significantly increased in the OPT, PS, and M treatments. In 2023, there was a significant increase in soluble sugars in the OPT, PS and M treatments. In 2022, sucrose levels significantly increased in the OPT, PS, and M treatments. However, in 2023, sucrose levels significantly increased in the OPT and PS treatments but remained unchanged in the M treatment. There were no significant differences in soluble sugar or glucose levels in 2022 or in glucose levels in 2023 between the OPT and PS or M treatments and the FP treatment. In terms of nutritional quality, compared with those in the FP treatment, vitamin C levels significantly increased in the OPT, PS, and M treatment groups in 2022. Conversely, in 2023, vitamin C levels significantly increased in the PS treatment but remained unchanged in the OPT and M treatments (**Figure 4A**). In 2022, the soluble protein content significantly increased in the OPT and PS treatments but remained unchanged in the M treatment (**Figure 4B**). Carotene significantly decreased in the M treatment but did not change in the OPT and PS treatments in 2022. In 2023, carotene significantly increased in the PS treatment but remained unchanged in the OPT and M treatments (**Figure 4C**). Free amino acids significantly increased in the PS treatment but did not change in the OPT and M treatments in 2022. In 2023, free amino acids significantly increased in the OPT and PS treatments but remained unchanged in the M treatment (**Figure 4D**).

**Table 3:** Effects of water and fertilizers management on the flavor quality in the upper, middle, lower segments in carrot.

Treatments/index			Total soluble solid (%)	Titratable acid (%)	TSS-to-titratable acid ratio	Soluble sugar (mg g^−1^ DW)	Glucose (mg g^−1^ DW)	Sucrose (mg g^−1^ DW)
Upper	2022	FP	8.10 ± 0.17b	0.58 ± 0.01a	14.55 ± 1.27b	351.22 ± 11.58a	116.29 ± 24.75ab	118.49 ± 12.28b
		OPT	8.17 ± 0.15ab	0.42 ± 0.06b	20.13 ± 2.57a	416.01 ± 14.42a	85.00 ± 37.88ab	191.36 ± 46.92a
		PS	8.47 ± 0.25a	0.43 ± 0.06b	20.37 ± 2.15a	448.86 ± 106.23a	139.42 ± 49.87a	176.95 ± 12.43a
		M	8.00 ± 0.00b	0.45 ± 0.11b	18.43 ± 3.90ab	422.37 ± 27.77a	66.98 ± 28.37b	221.54 ± 25.78a
	2023	FP	11.73 ± 0.15b	1.53 ± 0.04a	7.71 ± 0.33d	489.11 ± 5.11c	139.50 ± 100.16ab	149.36 ± 31.00b
		OPT	11.93 ± 0.15ab	1.21 ± 0.06b	9.94 ± 0.58c	608.60 ± 12.95b	83.62 ± 20.38b	229.50 ± 66.64a
		PS	12.20 ± 0.17a	1.00 ± 0.06c	12.29 ± 0.47a	682.72 ± 75.65ab	210.40 ± 66.60ab	265.45 ± 13.03a
		M	11.63 ± 0.25b	1.05 ± 0.01c	11.15 ± 0.15b	697.26 ± 42.59a	234.49 ± 60.42a	218.56 ± 14.58ab
Middle	2022	FP	7.87 ± 0.15b	0.74 ± 0.10a	10.88 ± 1.37b	400.91 ± 22.42b	132.09 ± 68.13a	222.47 ± 59.12a
		OPT	7.70 ± 0.10b	0.64 ± 0.05ab	12.28 ± 0.87b	441.06 ± 20.80b	208.85 ± 31.04a	208.22 ± 35.92a
		PS	8.33 ± 0.25a	0.55 ± 0.05b	16.07 ± 1.95a	548.61 ± 50.98a	104.68 ± 73.26a	247.57 ± 30.81a
		M	7.93 ± 0.12b	0.51 ± 0.07b	15.86 ± 1.99a	511.61 ± 16.96a	117.22 ± 36.08a	207.79 ± 55.06a
	2023	FP	11.37 ± 0.78a	1.54 ± 0.07a	7.41 ± 0.83b	650.25 ± 61.30a	279.26 ± 25.02a	159.18 ± 7.72b
		OPT	11.27 ± 0.35a	1.20 ± 0.05b	9.46 ± 00.62a	621.95 ± 36.74a	85.26 ± 29.6b	309.66 ± 71.02a
		PS	11.53 ± 0.12a	1.07 ± 0.15b	11.04 ± 1.67a	687.39 ± 44.23a	245.27 ± 20.14a	243.99 ± 4.58a
		M	11.50 ± 0.46a	1.08 ± 0.10b	10.64 ± 0.56a	676.24 ± 61.10a	232.05 ± 41.17a	243.12 ± 6.76a
Lower	2022	FP	7.60 ± 0.10b	1.18 ± 0.10a	6.53 ± 0.51c	459.33 ± 33.45c	201.58 ± 52.65a	122.59 ± 33.04b
		OPT	7.57 ± 0.25b	1.11 ± 0.06a	6.98 ± 0.50c	494.72 ± 18.33bc	72.05 ± 49.19b	229.54 ± 58.69a
		PS	8.13 ± 0.15a	0.95 ± 0.06b	8.61 ± 0.56b	518.26 ± 38.46b	127.77 ± 70.29ab	237.62 ± 27.60a
		M	7.93 ± 0.12a	0.74 ± 0.07c	10.78 ± 0.92a	575.84 ± 14.15a	199.76 ± 15.85a	145.84 ± 22.80b
	2023	FP	10.57 ± 0.29ab	1.48 ± 0.03a	7.18 ± 0.18c	468.86 ± 30.59c	168.87 ± 58.84b	102.61 ± 15.33c
		OPT	10.47 ± 0.15b	1.05 ± 0.11b	10.06 ± 1.13b	556.28 ± 29.51b	182.82 ± 4.26b	145.66 ± 11.36b
		PS	10.87 ± 0.21a	0.78 ± 0.01c	13.91 ± 0.18a	669.66 ± 33.31a	275.85 ± 20.35a	205.46 ± 19.08a
		M	10.40 ± 0.00b	0.81 ± 0.04c	12.86 ± 0.54a	676.07 ± 31.38a	281.30 ± 56.38a	155.80 ± 6.83b

Different letters indicate significant differences among different water and fertilizers managements (P < 0.05). Values shown are means (n = 3 replicates).

In terms of flavor quality in the middle carrot segment, the TSS content significantly increased in the PS treatment group compared with the FP treatment group but remained unchanged in the OPT and M treatment groups in 2022 (**Table 3**). Glucose levels significantly decreased in the OPT treatment, with no changes observed in the PS and M treatments in 2023. Additionally, in 2022, the TA significantly decreased, whereas the TSS-to-TA ratio and soluble sugars significantly increased in the PS and M treatments, but those parameters did not change in the OPT treatment. Furthermore, in 2023, the TA significantly decreased, whereas the TSS-to-TA ratio and sucrose content significantly increased in the OPT, PS, and M treatments. There were no significant differences in glucose or sucrose content in 2022, TSS content, or soluble sugar content among the OPT and PS or M treatments in 2023. In terms of nutritional quality, vitamin C significantly increased in the PS and M treatments but remained unchanged in the OPT treatment in 2022. In 2023, the vitamin C levels significantly increased in the OPT, PS, and M treatments (**Figure 4E**). The soluble protein levels significantly decreased in the OPT treatment, with no change observed in the PS and M treatments in 2022. Conversely, in 2023, the soluble protein content significantly decreased in the PS treatment, whereas no change was detected in the OPT and M treatments (**Figure 4F**). Carotene levels significantly increased in the OPT treatment but significantly decreased in the PS and M treatments in 2022. However, in 2023, carotene levels significantly increased in the PS treatment, with no change in the OPT and M treatments (**Figure 4G**). Free amino acid levels significantly increased in the OPT treatment, whereas no change was detected in the PS and M treatments in 2023. There were no significant differences in free amino acid levels in 2022 (**Figure 4H**).

In terms of flavor quality in the lower segment of carrots, compared with that in the FP treatment, the TSS significantly increased in the PS and M treatments but remained unchanged in the OPT treatment in 2022 (**Table 3**). The TA significantly decreased in the PS and M treatments but remained unchanged in the OPT treatment in 2022. In 2023, the TA significantly decreased in the OPT, PS, and M treatments. Glucose significantly decreased in the OPT treatment but remained unchanged in the PS and M treatments in 2022. In 2023, glucose significantly increased in the PS and M treatments but remained unchanged in the OPT treatment. Sucrose significantly increased in the OPT and PS treatments but remained unchanged in the M treatment in 2022. In 2023, sucrose significantly increased in the OPT, PS, and M treatments. The TSS-to-TA ratio and soluble sugar content significantly increased in the PS and M treatments but remained unchanged in the OPT treatment in 2022. In 2023, the TSS-to-TA ratio and soluble sugar content significantly increased in the OPT, PS, and M treatments. There were no significant differences in the TSS content between the OPT, PS, and M treatments and the FP treatment in 2023. In terms of nutritional quality, vitamin C significantly increased in the OPT, PS, and M treatments in both years (**Figure 4I**). The soluble protein content significantly decreased in the PS and M treatments but did not change in the OPT treatment in 2022 (**Figure 4J**). Carotene significantly increased in the OPT, PS, and M treatments in 2022 (**Figure 4K**). Free amino acids significantly increased in the OPT and PS treatments but remained unchanged in the M treatment in 2022 (**Figure 4L**). No significant differences were found in soluble protein, carotene or free amino acids in 2023 (**Figures 4J–L**).

### 3.3 Flavor and nutritional quality in the phloem and xylem

**Figure 5:**
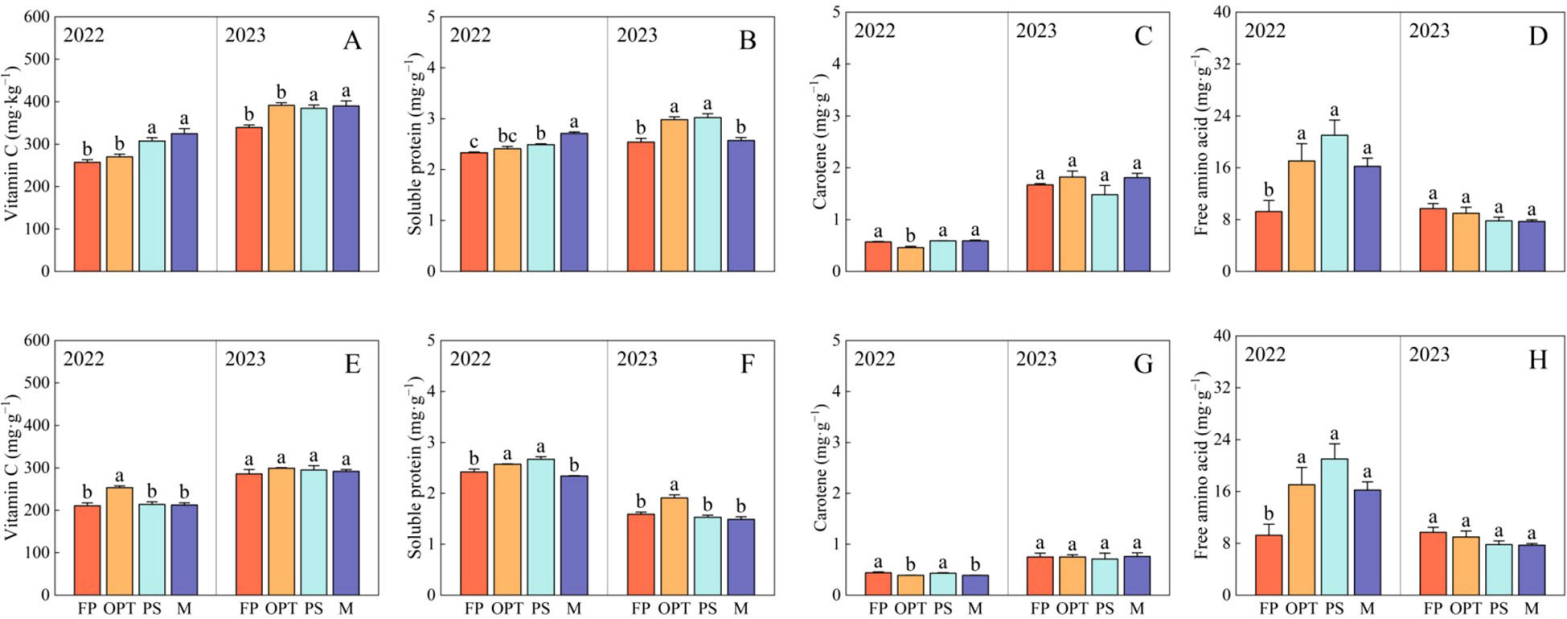
Effects of water and fertilizers management on the nutritional quality of phloem and xylem in carrot. Different letters indicate significant differences among different water and fertilizers managements (P < 0.05). Values shown are means (n = 3 replicates). Different letters indicate significant differences among different water and fertilizers managements (P < 0.05). Values shown are means (n = 3 replicates). **(A–D)** represent nutritional quality in the phloem of FP, OPT, PS, and M treatments, respectively. **(E–H)** represent nutritional quality in the xylem of FP, OPT, PS, and M treatments of FP, OPT, PS, and M treatments, respectively.

Carrots were separated into phloem and xylem to assess flavor and nutritional quality on the basis of their radial distribution (**Figure 1**). The flavor and nutritional quality of the phloem and xylem are similar to the growth patterns of the whole fleshy roots (**Table 4**; **Figure 5**). Compared with that in the FP treatment with respect to flavor quality of the phloem in 2022, TSS significantly increased in the PS treatment but remained unchanged in the OPT and M treatments (**Table 4**). The TA and glucose contents significantly decreased in the M treatment, whereas no changes were detected in the OPT and PS treatments. The TSS-to-TA ratio significantly increased in the M treatment but did not change in the OPT and PS treatments. The soluble sugar content significantly increased in the OPT treatment but remained unchanged in the PS and M treatments. The sucrose content significantly increased in the PS and M treatments, whereas no changes were detected in the OPT treatment. In terms of flavor quality in the phloem in 2023, compared with those in the FP treatment, the contents of TSS and TA significantly decreased in the M treatment, with no changes in the OPT and PS treatments. The TSS-to-TA ratio significantly increased in the M treatment, with no changes in the OPT and PS treatments. Glucose levels significantly decreased in the PS and M treatments but remained unchanged in the OPT treatment. Sucrose levels significantly increased in the PS and M treatments but remained unchanged in the OPT treatment. There were no significant differences in soluble sugar content in 2023. In terms of flavor quality in the xylem in 2022, compared with those in the FP treatment, the contents of TSS, soluble sugars, and sucrose significantly increased in the PS and M treatments but remained unchanged in the OPT treatment (**Table 4**). Compared with that in the FP treatment, the flavor quality of the xylem in the OPT, PS, and M treatments significantly improved in terms of the TSS-to-TA ratio in 2023. Additionally, the TA significantly decreased in the OPT, PS, and M treatments in 2023. The soluble sugar levels significantly increased in the PS and M treatments but did not change in the OPT treatment. There were no significant differences observed in TA, TSS-to-TA, or glucose concentrations in 2022 or in TSS or glucose concentrations in 2023.

**Table 4:** Effects of water and fertilizers management on the flavor quality of phloem and xylem in carrot.

Treatments/index			Total soluble solid (%)	Titratable acid (%)	TSS-to-titratable acid ratio	Soluble sugar (mg g^−1^ DW)	Glucose (mg g^−1^ DW)	Sucrose (mg g^−1^ DW)
2022	Phloem	FP	8.13 ± 0.06b	0.54 ± 0.14a	15.97 ± 4.44b	475.10 ± 28.73b	130.33 ± 14.10ab	184.96 ± 15.27b
		OPT	8.37 ± 0.15b	0.49 ± 0.00ab	17.92 ± 0.58ab	545.68 ± 30.39a	172.49 ± 49.68a	197.21 ± 35.96b
		PS	8.70 ± 0.26a	0.43 ± 0.06ab	20.94 ± 2.73ab	513.34 ± 9.48ab	68.88 ± 48.37bc	251.90 ± 25.94a
		M	8.20 ± 0.00b	0.36 ± 0.05b	24.77 ± 6.08a	507.99 ± 28.87ab	33.72 ± 27.74c	275.89 ± 14.88a
	Xylem	FP	7.63 ± 0.12b	1.27 ± 0.11a	6.02 ± 0.45a	365.47 ± 32.54c	96.84 ± 29.55a	162.85 ± 6.79c
		OPT	7.70 ± 0.10b	1.20 ± 0.06a	6.49 ± 0.43a	397.74 ± 32.90bc	70.50 ± 23.49a	162.41 ± 2.04c
		PS	8.13 ± 0.12a	1.21 ± 0.07a	6.72 ± 0.30a	445.23 ± 27.36b	53.03 ± 13.07a	224.78 ± 26.51b
		M	7.93 ± 0.12a	1.20 ± 0.06a	6.74 ± 0.40a	509.32 ± 27.64a	90.85 ± 38.72a	259.01 ± 8.91a
2023	Phloem	FP	12.43 ± 0.21a	1.14 ± 0.04a	10.97 ± 0.29b	696.18 ± 25.93a	303.72 ± 27.96a	143.12 ± 1.75b
		OPT	12.53 ± 0.21a	1.09 ± 0.06a	11.53 ± 0.58ab	745.63 ± 27.64a	342.86 ± 33.58a	152.38 ± 7.69b
		PS	12.67 ± 0.15a	1.08 ± 0.01a	11.81 ± 0.05ab	719.19 ± 31.68a	196.64 ± 29.85b	275.57 ± 10.95a
		M	11.87 ± 0.32b	0.97 ± 0.08b	12.32 ± 0.81a	753.26 ± 44.81a	163.53 ± 34.35b	332.07 ± 61.09a
	Xylem	FP	10.53 ± 0.31a	2.50 ± 0.16a	4.24 ± 0.39d	490.98 ± 34.63c	188.00 ± 22.59a	134.10 ± 19.19b
		OPT	10.53 ± 0.15a	1.91 ± 0.06b	5.51 ± 0.19c	526.93 ± 18.48bc	123.99 ± 54.74a	130.22 ± 9.22b
		PS	10.40 ± 0.10a	1.71 ± 0.02c	6.09 ± 0.01b	584.24 ± 35.73ab	120.65 ± 41.94a	215.04 ± 5.31a
		M	10.20 ± 0.00a	1.54 ± 0.02c	6.61 ± 0.10a	607.22 ± 34.12a	146.39 ± 16.42a	192.41 ± 13.25a

Different letters indicate significant differences among different water and fertilizers managements (P < 0.05). Values shown are means (n = 3 replicates).

In terms of nutritional quality in the phloem, compared with that in the FP treatment, vitamin C significantly increased in the PS and M treatments but remained unchanged in the OPT treatment in both years (**Figure 5A**). The soluble protein content significantly increased in the PS and M treatments but remained unchanged in the OPT treatment in 2022. However, in 2023, the soluble protein content significantly increased in the OPT and PS treatments, but remained unchanged in the M treatment (**Figure 5B**). Carotene significantly decreased in the OPT treatment but remained unchanged in the PS and M treatments in 2022 (**Figure 5C**). Free amino acids significantly increased in the OPT, PS, and M treatments in 2022 (**Figure 5D**). There were no significant differences in carotene or free amino acids in 2023 (**Figures 5C, D**). In terms of nutritional quality in the xylem, compared with that in the FP treatment, vitamin C significantly increased in the OPT treatment but remained unchanged in the PS and M treatments in 2022 (**Figure 5E**). In 2022, the soluble protein content significantly increased in the OPT and PS treatments but remained unchanged in the M treatment. Conversely, in 2023, the soluble protein content significantly increased in the OPT treatment but remained unchanged in the PS and M treatments (**Figure 5F**). Carotene levels significantly decreased in the OPT and M treatments, with no significant change in the PS treatment in 2022 (**Figure 5G**). Free amino acid levels significantly increased in the OPT, PS, and M treatments in 2022 (**Figure 5H**). However, there were no significant differences in vitamin C, carotene or free amino acid contents in 2023 (**Figures 5E–H**).

**Figure 6:**
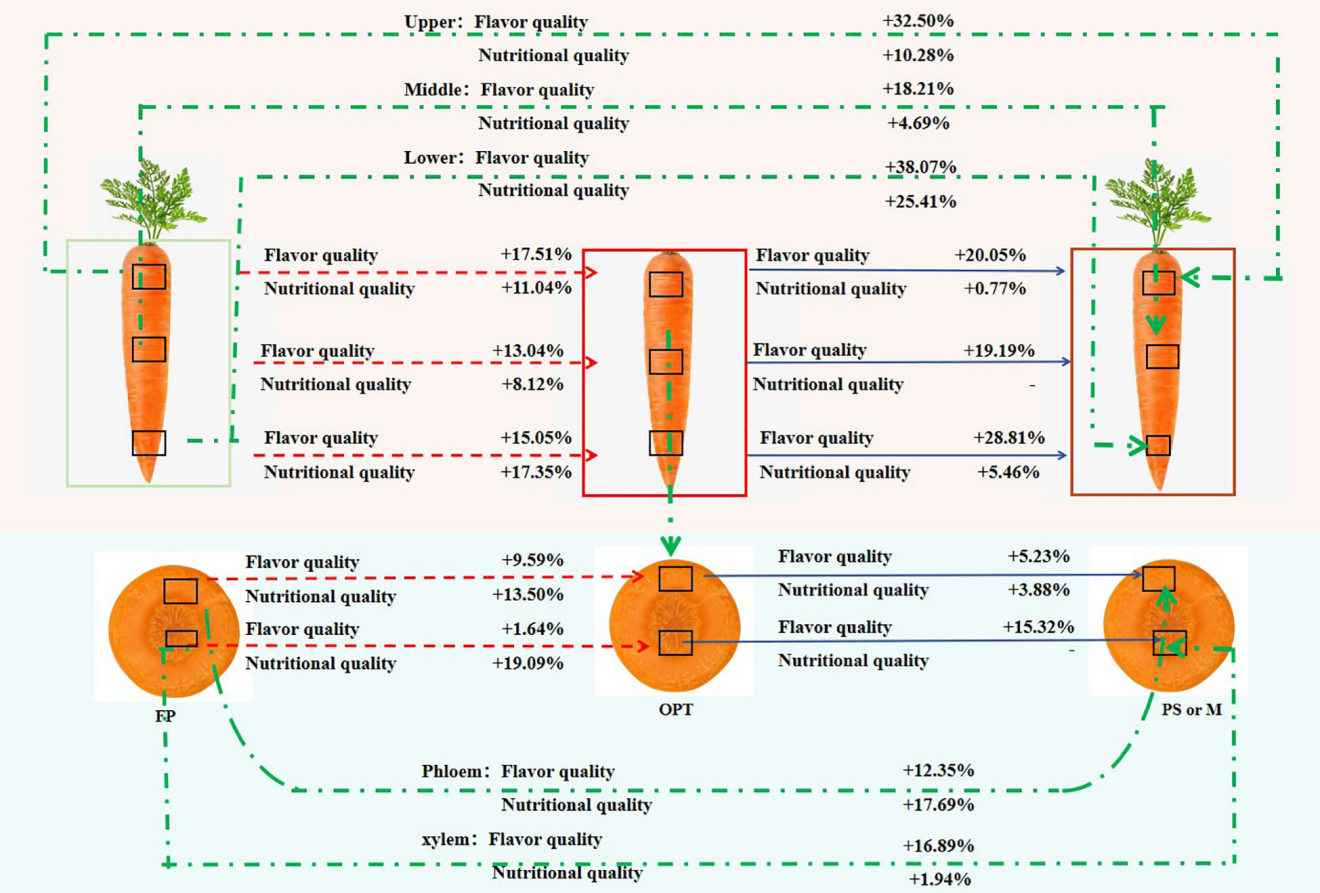
The changes of quality from farmers’ habit (FP) to coordinated water-fertilizers management (OPT, PS, or M) in the upper, middle and lower segments and phloem and xylem in carrot. + indicates the percentage of quality improvement, and - indicates no difference. The red background indicates the changes of quality in the upper, middle and lower segments. The blue background indicates the changes of quality in phloem and xylem. The red line indicates the changes of quality the FP treatment to OPT treatment in the upper, middle and lower segments and phloem and xylem. The blue line indicates the changes of quality the OPT treatment to PS or M treatment in the upper, middle and lower segments and phloem and xylem. The green line indicates the changes of quality the FP treatment to PS or M treatment in the upper, middle and lower segments and phloem and xylem.

Overall, compared with that in the FP treatment, the flavor quality increased by 17.51%, 13.04%, and 15.05% in the upper, middle, and lower segments, respectively, whereas the nutritional quality increased by 11.04%, 8.12%, and 17.35%, respectively, in the OPT treatment (**Figure 6**). In the organic substitution treatments (average of PS and M), the flavor quality increased by 32.50%, 18.21%, and 38.07%, and the nutritional quality increased by 10.28%, 4.69%, and 25.41%, respectively. Compared with those in the OPT treatment, the flavor and nutritional quality of all the segments in the PS and M treatments improved, with the most significant increase observed in the lower segment. Specifically, the flavor quality increased by 20.05%, 19.19%, and 28.81% in the upper, middle, and lower segments, respectively, while the nutritional quality increased by 0.77% and 5.46% in the upper and lower segments, respectively, under the organic substitution treatments. These findings indicate that the enhancements in flavor and nutritional quality of whole carrot roots are due mainly to improvements in the upper and lower segments under coordinated water and organic–inorganic fertilizer management. In the phloem, flavor and nutritional quality increased by 9.59% and 13.50%, respectively, in the OPT treatment compared with the FP treatment and by 12.35% and 17.69%, respectively, in the organic substitution treatment. In the xylem, flavor and nutritional quality increased by 1.64% and 19.09%, respectively, in the OPT treatment, whereas flavor quality increased by 16.89% and 1.94%, respectively, in the organic substitution treatment. Compared with the OPT treatment, the flavor and nutritional quality of the phloem increased by 5.23% and 3.88%, respectively, in the organic substitution treatment. These results suggest that enhancing the flavor and nutritional quality of both the phloem and xylem can improve the overall flavor and nutritional quality of the entire fleshy root in terms of radial distribution under coordinated water and organic–inorganic fertilizer management.

### 3.4 Anatomical structure of the phloem and xylem in the upper, middle, and lower segments

**Figure 7:**
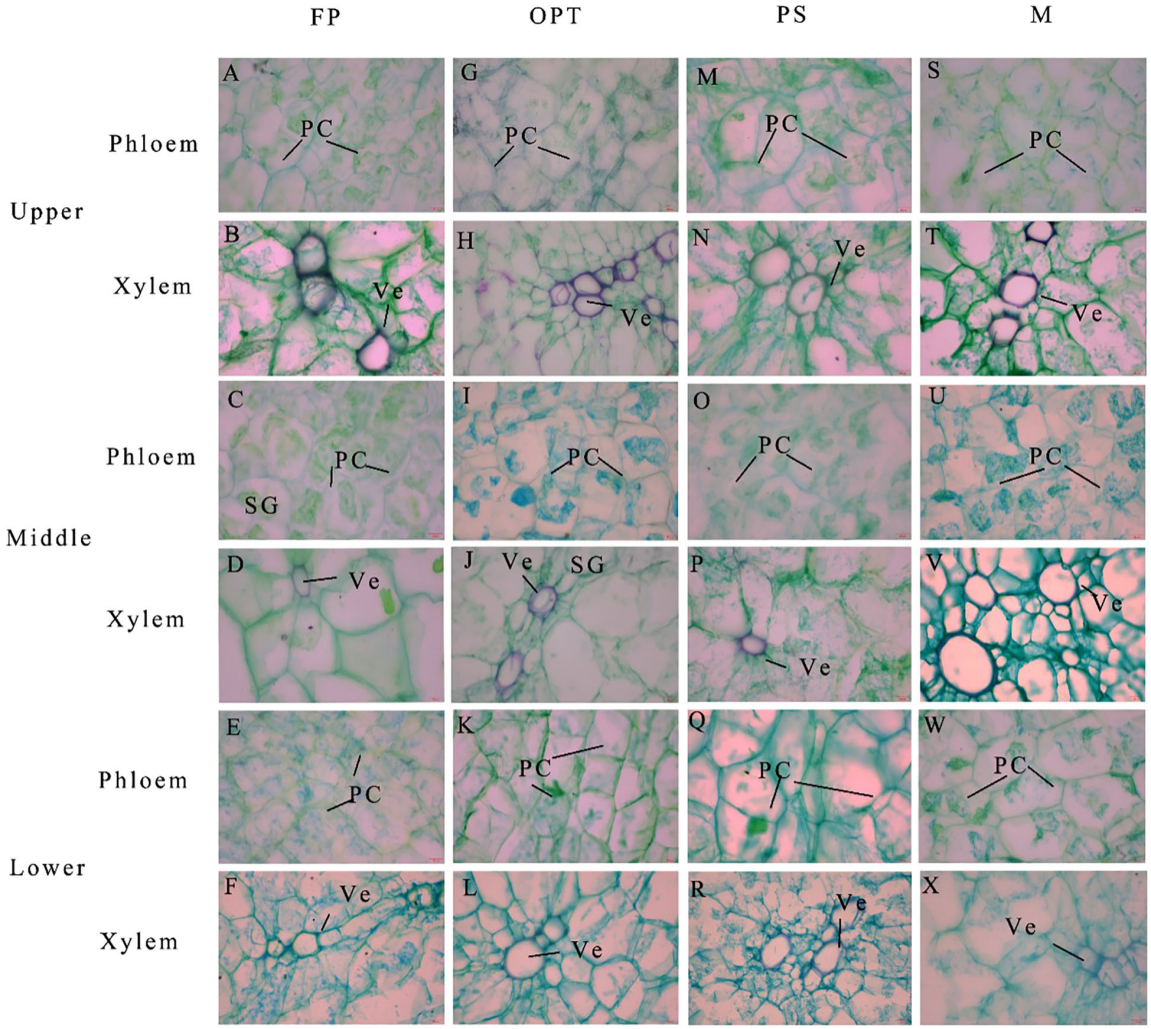
Effects of water and fertilizers management on the anatomical structure of phloem and xylem in carrot. **(A, C, E)** and **(G, I, K)** and **(M, O, Q)** and **(S, U, W)** represent the cross sections of phloem in the FP, OPT, PS, and M treatments in the upper, middle and lower segments, respectively. **(B, D, F)** and **(H, J, L)** and **(N, P, R)** and **(T, V, X)** represent the cross sections of xylem in the FP, OPT, PS, and M treatments in the upper, middle and lower segments, respectively. Parenchymal cell (PC), starch granule (SG) and vessel (Ve) are marked. Scale bars are 20 mm length in all the figures.

**Figure 8:**
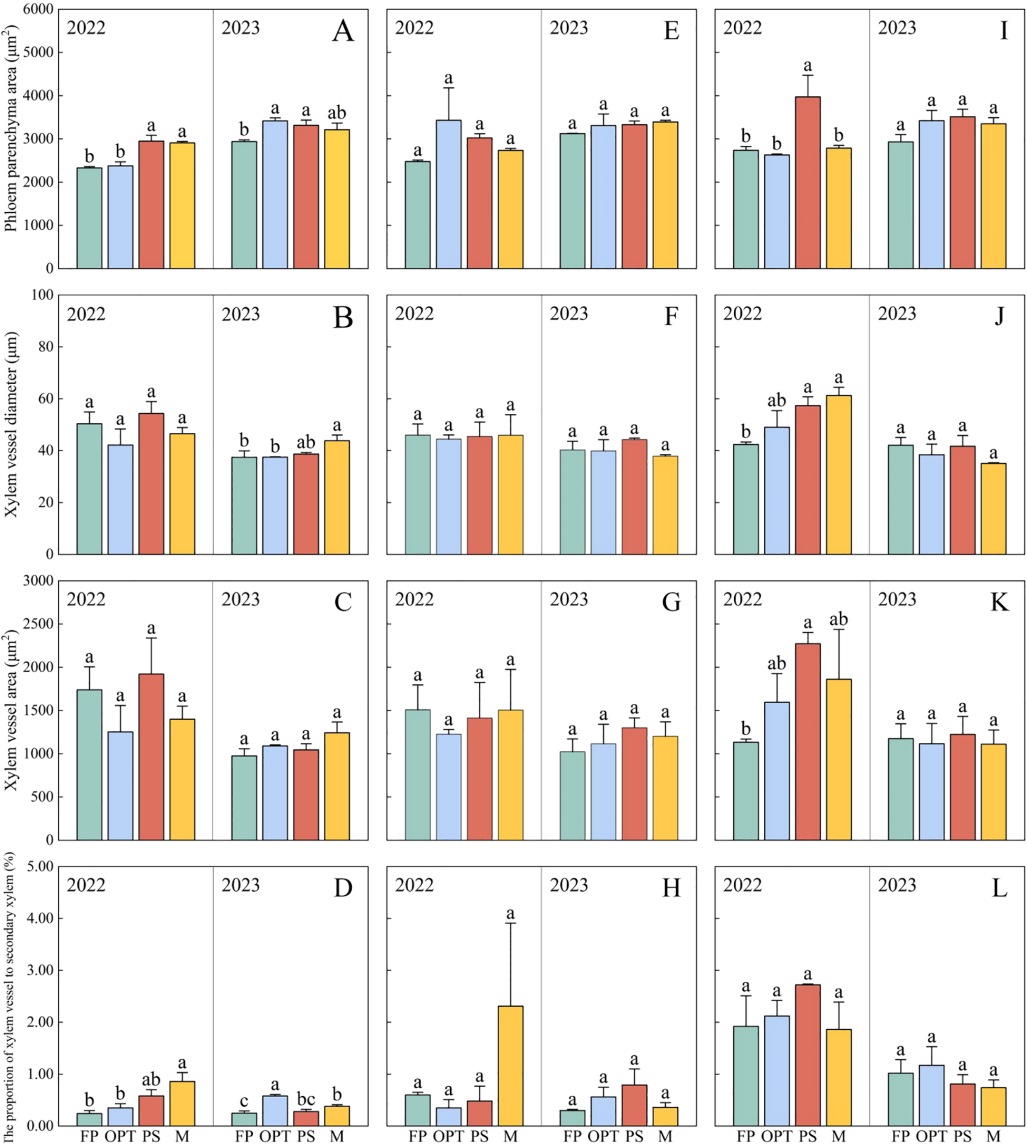
Effects of water and fertilizers management on the anatomical structure of phloem and xylem in carrot. **(A–D)** represent parenchyma cell area, xylem vessel diameter, xylem vessel area, the proportion of xylem vessel to secondary xylem of FP, OPT, PS, and M treatments, in the upper segment, respectively. **(E–H)** represent parenchyma cell area, xylem vessel diameter, xylem vessel area, the proportion of xylem vessel to secondary xylem of FP, OPT, PS, and M treatments, in the middle segment, respectively. **(I–L)** represent parenchyma cell area, xylem vessel diameter, xylem vessel area, the proportion of xylem vessel to secondary xylem of FP, OPT, PS, and M treatments, in the lower segment, respectively. Data are the means for the 2 years. Different letters indicate significant differences among different water and fertilizers managements (P < 0.05). Values shown are means (n = 3 replicates).

In the upper segment, Pca significantly increased in the PS and M treatments compared with the FP treatment but remained unchanged in the OPT treatment in 2022 (**Figures 7**, **8A**). In 2023, Pca significantly increased in the OPT and PS treatments, whereas no change was detected in the M treatment. In 2023, the xylem vessel diameter (Xvd) significantly increased in the M treatment (**Figures 7**, **8B**). The Pxv significantly increased in the M treatment in 2022 (**Figures 7**, **8C**). In 2023, the Pxv significantly increased in the OPT and M treatments but remained unchanged in the PS treatment (**Figures 7**, **8D**). No significant differences were detected in Xvd or the xylem vessel area (Xva) in 2022 or in Xva in 2023 (**Figures 7**, **8B, C**). In the middle segment, no significant differences were found in Pca, Xvd, Xva, or Pxv among the four treatments (**Figures 7**, **8E–H**). Compared with that in the FP treatment, the Pca in the lower segment significantly increased in the PS treatment but remained unchanged in the OPT and M treatments in 2022 (**Figure 8I**). Xvd significantly increased in the PS and M treatments but remained unchanged in the OPT treatment in 2022 (**Figure 8J**). In 2022, Xva significantly increased in the PS treatment but remained unchanged in the OPT and M treatments (**Figure 8K**). No significant differences were found in Pxv in 2022 or in Pca, Xvd, Xva, or Pxv in 2023 (**Figures 8E–H**). These results suggest that the primary distinctions between the FP treatment and optimized water-fertilizer management lie in Pca and Pxv in the upper segment.

### 3.5 Correlations between quality and the anatomical structural characteristics of the upper, middle, and lower segments of the phloem and xylem

**Figure 9:**
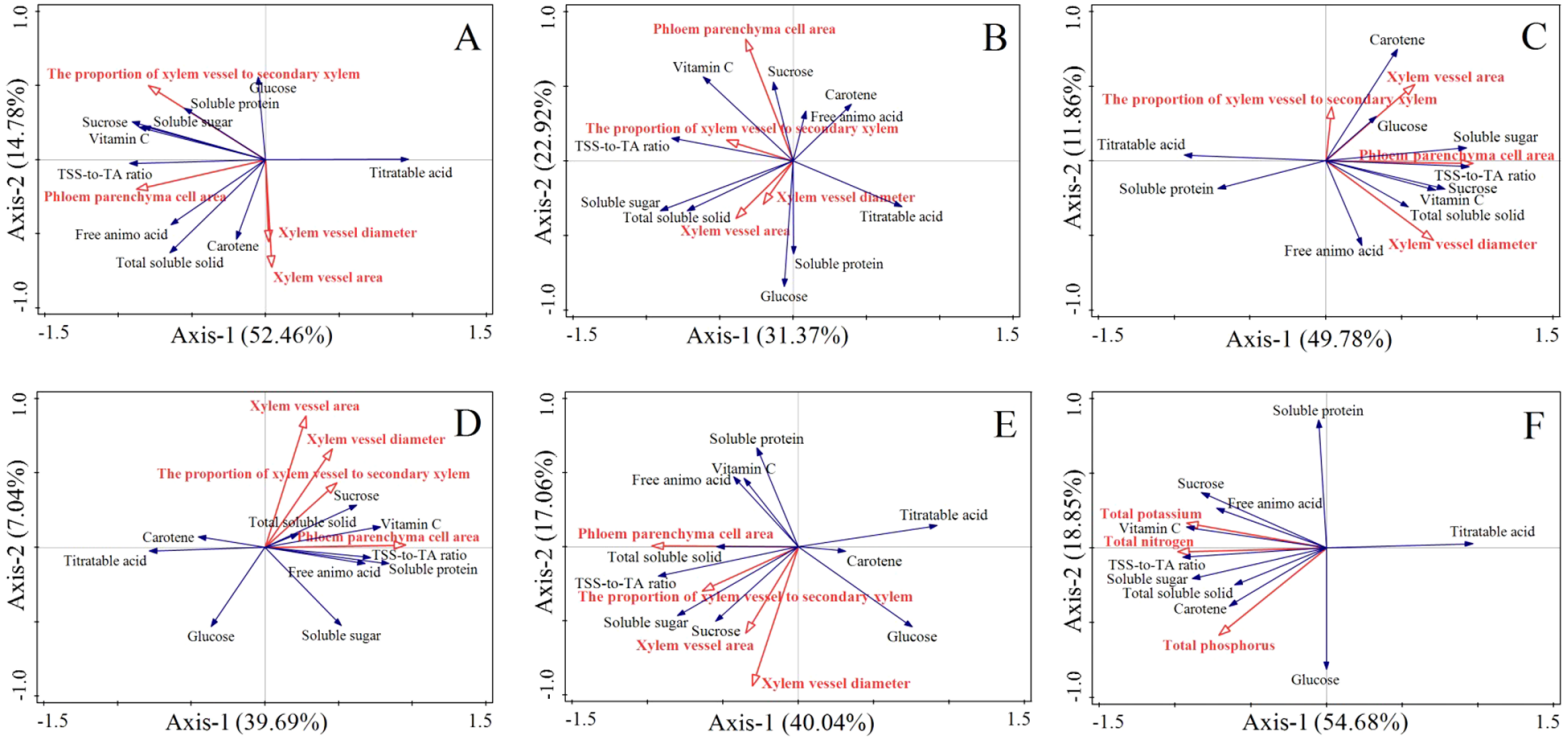
Independent and interactive actions of phloem parenchyma cell area, xylem vessel diameter, xylem vessel area, the proportion of xylem vessel to secondary xylem and total nitrogen, phosphorus, total potassium on various properties shown in ordination diagrams obtained from redundancy analysis (RDA). **(A-E)** represent RDA analysis in the upper, middle, lower segment, phloem and xylem of carrots in the FP, OPT, PS, or M treatment, respectively. The coordinate of the first and second ordination axes explained 52.46% and 14.78% of the variance in **(A)** 31.37% and 22.92% of the variance in **(B)** 49.78% and 11.86% of the variance in **(C)** 39.69% and 7.04% of the variance in **(D)** 40.04% and 17.06% of the variance in **(E)**. **(F)** represent RDA analysis of carrots in the FP, OPT, PS, or M treatment, respectively. The coordinate of the first and second ordination axes explained 54.68% and 18.85% of the variance in F.

**Figure 10:**
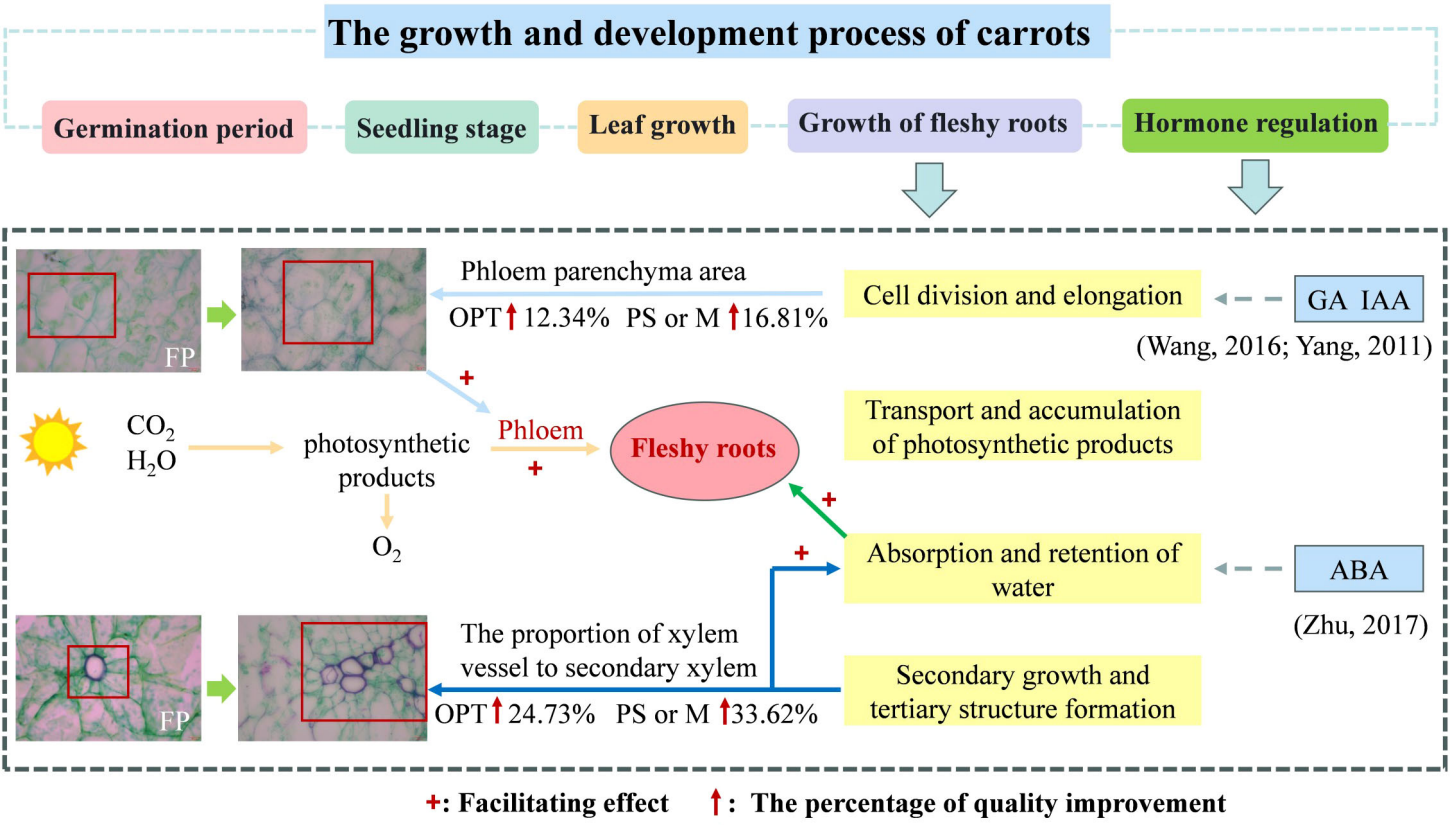
Physiological changes of carrot in optimizing water and fertilizer management compared to farmers practice. + indicates facilitation. ↑indicates the percentage of quality improvement compared to FP.

In the upper segment, Pca and Pxv were significantly correlated with flavor quality and nutritional quality (P<0.05), whereas Xva and Xvd were not correlated (**Figure 9A**). Specifically, Pca was positively correlated with soluble protein, sucrose, soluble sugars, vitamin C, the TSS-to-TA ratio, free amino acids, TSS, and carotene. Pxv was positively correlated with glucose, soluble protein, soluble sugar, sucrose, vitamin C, the TSS-to-TA ratio, free amino acids and TSS. However, no significant correlations were detected in the middle segment (**Figure 9B**). In the lower segment, Pca was significantly correlated with flavor quality and nutritional quality (P<0.05), whereas Pxv, Xva, and Xvd were not significantly correlated (**Figure 9C**). Pca was positively correlated with carotene, glucose, soluble sugars, the TSS-to-TA ratio, vitamin C, sucrose, TSS, and free amino acids. In the phloem, Pca was significantly correlated with flavor quality and nutritional quality (P<0.05), whereas Pxv, Xvd, and Xva were not significantly correlated (**Figure 9D**). Pca was positively correlated with TSS, sucrose, vitamin C, soluble protein, the TSS-to-TA ratio, free amino acids, and soluble sugars. Within the xylem, Pca was significantly correlated with flavor quality and nutritional quality (P<0.05), whereas Pxv, Xvd, and Xva were not significantly correlated (**Figure 9E**). Pca was positively correlated with soluble protein, free amino acids, vitamin C, TSS, the TSS-to-TA ratio, soluble sugars, and sucrose. These findings indicate that the increase in quality in the upper segment was driven primarily by improvements in Pca and Pxv, whereas the increase in quality in the lower segment was associated predominantly with an increase in Pca (**Figure 10**). The improvement in the quality of the phloem and xylem was attributed mainly to the increase in Pca.

### 3.6 Correlations between the quality and nutrient concentration of whole fleshy roots

The concentrations of N and P were correlated with both flavor quality and nutritional quality (P<0.05) (**Figure 9F**). Specifically, the N concentration was positively correlated with soluble protein, free amino acids, sucrose, VC, the TSS-to-TA ratio, soluble sugars, TSS, carotene, and glucose. Similarly, the P concentration was positively correlated with sucrose, free amino acids, VC, the TSS-to-TA ratio, soluble sugars, TSS, carotene, and glucose. These results suggest that the nutrient levels of N and P may influence carrot quality.

## 4 Discussion

### 4.1 Role of coordinated water and fertilizer management on yield and quality

Coordinated water-fertilizer management plays a key role in vegetable yield and quality (Lin et al., 2020). Efficient irrigation is essential for promoting crop growth, especially considering that the response of crops to moisture levels directly impacts their physiological and metabolic functions (He et al., 2021). The incorporation of water-fertilizer technology led to a 20% reduction in nitrogen (N), phosphorus (P), and potassium (K) fertilizers, resulting in increased yield, quality, and fertilizer utilization in tomato (Huang and Chen, 2020). Compared with those under the FP treatment, the yield and quality of whole fleshy roots significantly increased under coordinated water-fertilizer management, particularly when 30% of the chemical fertilizers were substituted with compressed organic fertilizers (**Table 2**; **Figures 2**, **3**). This finding aligns with those of previous studies. Accurate management of integrated water and fertilizer is crucial for growth regulation, improving efficient water-fertilizer utilization, and achieving high yields (Liu et al., 2021). The combination of water and fertilizer can improve the ecological environment and the soil nutrient content (Liu et al., 2023). The appropriate regulation of water and fertilizer can increase leaf area and root growth, thereby increasing crop water and nutrient uptake through osmoregulation and antioxidant capacity (Zhao et al., 2022). Various irrigation and fertilization rates result in differences in root uptake efficiency, impacting root growth, the root–soil contact area, and ultimately, yield (Chen et al., 2001). In this study, the yields of the four treatments in 2022 varied significantly (**Figure 2**). This could be due to the increase in local precipitation after carrot sowing, which caused excessive soil moisture that hindered the complete germination of certain carrot seeds. Additionally, the use of different carrot varieties may have contributed to yield discrepancies between the two years, ultimately impacting the overall carrot yield.

This study confirmed that carrot quality can be enhanced with PS or M treatment (**Table 2**; **Figure 3**). A previous study revealed that replacing 25–50% of inorganic N, P, and K fertilizer with organic modifiers led to the highest nutritional quality of food crops (Ishfaq et al., 2023). The substitution of 25% N fertilizer with organic fertilizer significantly affects the levels of vitamin C, soluble sugar, soluble protein, and nitrate in cucumbers (Zhang, 2023). Organic fertilizers contain sufficient nutrients and release them slowly, thereby improving crop yield and quality to different extents (Jiang et al., 2021). Organic substitution can increase soil bacterial and fungal biomass, thereby improving soil physicochemical conditions and fertility (Zhao et al., 2023). Organic fertilizers not only provide essential nutrients for crops but also harbor microorganisms that improve the soil environment (Pang, 2023). Nevertheless, compared with OPT, organic substitution has limited efficacy in enhancing the appearance, flavor, and nutritional quality of carrots (**Table 2**; **Figure 3**). This is possibly due to the slow-release nature of fertilizers in organic substitution and the relatively short duration of the substitution, hindering the complete exploitation of the long-term benefits of organic amendments.

### 4.2 The contribution of the quality of different segments to the overall quality of the fleshy root

N, P, and K are crucial for biomass allocation and root elongation in tubers; thus, regulating assimilate allocation is also essential (Cakmak et al., 1994). This phenomenon may, in turn, affect the quality of the upper, middle, and lower segments of root crops. These findings were consistent with the results that the concentrations of N and P were correlated with both flavor quality and nutritional quality (P<0.05) (**Figure 9F**). Moreover, in root crops, the number of root tips determines the efficiency of water and nutrient absorption (Yuan et al., 2023). Previous studies have focused on the assimilation processes of both aboveground and underground plant parts (Cheng et al., 2023). In this study, the flavor quality and nutritional quality of carrots were improved under coordinated water-fertilizer management (OPT, PS, or M treatments), particularly in the upper and lower plant parts (**Table 3**; **Figures 4**, **6**). The superior quality in the upper segment could be attributed to the shorter distance between the aboveground parts, leading to an increased ion concentration gradient between the soil and roots, facilitating diffusion, and thereby improving quality (Barber, 1995). The quality of the lower segment may improve due to an increase in the number of carrot root tip cells, likely because of the presence of more ultrastructures (such as the endoplasmic reticulum, mitochondria, Golgi apparatus, ribosomes, vacuoles, microsomes, and plasma membrane ATPases) in the root tip cells that are vital for root function (Yang et al., 2012). Plant root tip cells may undergo structural damage and organelle deformation in cases of nutrient deficiencies, such as phosphorus, zinc, and silicon deficiencies (Miras et al., 2022), resulting in hindered nutrient transport to the roots (Dinar and Rudich, 1985). Compared with N in surface soil, N in deep soil is prone to leaching (Tei et al., 2020). In this study, optimizing water-fertilizer management, especially in the PS or M treatments, provided nutrients and water at appropriate times to improve root morphological indices (root tips) and improve quality in the lower segment (**Table 3**; **Figures 4**, **6**).

This study revealed a significant improvement in the nutritional quality of soluble sugars, sucrose, and vitamin C in the upper, middle, and lower segments (**Table 3**; **Figures 4**, **6**). Within the framework of integrated water-fertilizer management, this mechanism facilitates the translocation of carbohydrates from vegetative tissues to storage organs, potentially increasing the enzymatic activity of Frk and starch synthase, along with sucrose levels (Hennion et al., 2019). Munch’s hypothesis suggests that the flow of solution through the phloem is driven by a hydrostatic pressure gradient (Winfried and Michael, 2022). Many plant growth regulators, including auxins, gibberellins, humic acids, and cytokinins derived from microorganisms, are rich in organic fertilizers, thereby increasing vitamin C (Zuo et al., 2018). As a result, the nutritional quality significantly improved in the upper, middle, and lower segments under coordinated water-fertilizer management.

### 4.3 The effects of the anatomical structure of the phloem and xylem on quality

The taproot of the root tubers comprises two primary regions: the outer ring of the phloem parenchyma and the inner core of the xylem parenchyma. These regions originate from a cambium formed between the xylem and phloem vessels in seedling roots (Liu et al., 2022). Vascular parenchyma cell activity in the root is crucial for metabolic and energy connections, supporting water and nutrient transport in the xylem and facilitating both short- and long-distance symbiotic transport (Spicer, 2014). Expanding the area of ducts, xylem, and phloem establishes a strong transport network that optimizes the water supply, guaranteeing regular growth and development (Wang et al., 2023). Various abiotic stresses, such as N deficiency and temperature, influence the size and density of parenchyma, thereby compromising water and nutrient transportation among organs and affecting quality (Ren et al., 2021). In this study, Pca and Pxv in the upper segment of the OPT, PS, or M treatments were 9.17%, 88.40%, and 18.44%, 116.22% greater, respectively, than those in the FP treatment (**Figures 7**, **8**, **10**). Zhao and Gu (1999) reported that an increased volume of parenchymatous cells improved the nutritional quality of carrots. Furthermore, RDA revealed a positive correlation between Pca and Pxv levels in the upper segment and flavor as well as nutritional quality (**Figure 9**). Under coordinated water-fertilizer management, isocompounds typically move faster from the active phloem outward (centrifugally) than inward (Korolev et al., 2000). The increased growth of phloem parenchyma tissue and carbon deposition may increase the competitiveness of growing organs for available photosynthates, possibly because of their unique transport characteristics within the carrot taproot (Wareing and Patrick, 1975). This structure supports sucrose accumulation with low glucose content in the taproot (Liu et al., 2018). Vacuoles in phloem parenchyma cells store nutrients, such as soluble sugars, thereby improving carrot quality. The optimized water-fertilizer management in this study resulted in an increased Pca and vessel proportions distributed across the xylem region, potentially promoting xylem tissue expansion (**Figure 10**). Studies indicate that cell size influences sucrose absorption in beets (Hoffmann, 2010). The radish taproot xylem vessels, which are radially distributed but not densely packed, facilitate parenchyma development (Feng et al., 2017). The improvement in flavor and nutritional quality of both the upper and lower segments is likely linked to elevated Pca and Pxv levels. Carrots with relatively high Pca and Pxv values presented improved flavor and nutritional quality. It has been demonstrated that gibberellic acid (GA) regulates root growth by promoting cell elongation and controlling cell number (Inada and Shimmen, 2000; Shani et al., 2013; Ubeda-Tomás et al., 2009). IAA stimulates the formation of lateral root primordia, and the occurrence and elongation of lateral roots depend on the direct or indirect regulatory effects of growth hormone (Kazan, 2013). ABA can help plants adapt to diverse environments by regulating the growth and development of primary and lateral roots, thereby indirectly aiding in anchoring plants and facilitating water and nutrient absorption (Zhu et al., 2017). However, this study did not delve deeply into the effects of phytohormones. Therefore, further investigation into the influence of phytohormones on the expansion of carrot fleshy roots is warranted in future research.

## 5 Conclusion

Compared with traditional farming practices, coordinated water-fertilizer management can increase both quality and yield, with 30% organic substitution resulting in superior results. This improvement in quality is attributed to improved flavor and nutritional characteristics in the upper and lower segments, as well as in the phloem and xylem tissues. High-quality carrots present increased phloem parenchyma cells and a greater proportion of xylem vessels. Therefore, improving the flavor and nutritional quality of the upper and lower segments could serve as an effective approach to improve the overall quality of high-yield carrot. The phloem parenchyma cell area and xylem vessel proportion are potentially valuable physiological traits for optimizing quality. Additionally, further research is needed to investigate the impact of phytohormones on the growth of carrot fleshy roots.

## Data Availability

The original contributions presented in the study are included in the article/**Supplementary Material**. Further inquiries can be directed to the corresponding author.
