# Supplementary material for: High-quality carrots in relation to the superior phloem parenchyma cells and proportion of xylem vessel in coordinated water-fertilizer management
Source: Front Plant Sci. 2025 Jun 13;16:1590774. doi: 10.3389/fpls.2025.1590774 (PMC12202603; doi:10.3389/fpls.2025.1590774)
Supplement: Supplementary file 1 [file DataSheet1.docx]

Supplementary Material

# Supplementary Tables

**Table S1. Nutrient input at different growth stages under each treatment in autumn 2022 and spring 2023.**

| Year | | the base stage | | | the seeding stage | | | the prerosette stage | | | the rosette stage | | | the early succulent root enlargement | | | the succulent root expansion stage | | | the fleshy root enlargement metaphase | | |
| --- | --- | --- | --- | --- | --- | --- | --- | --- | --- | --- | --- | --- | --- | --- | --- | --- | --- | --- | --- | --- | --- | --- |
|  |  | N | P_2_O_5_ | K_2_O | N | P_2_O_5_ | K_2_O | N | P_2_O_5_ | K_2_O | N | P_2_O_5_ | K_2_O | N | P_2_O_5_ | K_2_O | N | P_2_O_5_ | K_2_O | N | P_2_O_5_ | K_2_O |
|  |  | (kg ha^-1^) | (kg ha^-1^) | (kg ha^-1^) | (kg ha^-1^) | (kg ha^-1^) | (kg ha^-1^) | (kg ha^-1^) | (kg ha^-1^) | (kg ha^-1^) | (kg ha^-1^) | (kg ha^-1^) | (kg ha^-1^) | (kg ha^-1^) | (kg ha^-1^) | (kg ha^-1^) | (kg ha^-1^) | (kg ha^-1^) | (kg ha^-1^) | (kg ha^-1^) | (kg ha^-1^) | (kg ha^-1^) |
| 2022 Autumn | FP | 273.0 | 225.0 | 225.0 | 22.5 | 0.0 | 7.5 | - | - | - | - | - | - | 27.0 | 0.0 | 52.5 | - | - | - | - | - | - |
|  | OPT | 132.0 | 36.0 | 120.0 | 26.4 | 2.7 | 18.0 | 26.4 | 2.7 | 18.0 | 26.4 | 2.7 | 18.0 | 31.8 | 5.4 | 36.0 | 9.0 | 4.5 | 30.0 | - | - | - |
|  | PS | 132.0 | 45.0 | 120.0 | 26.4 | 2.7 | 18.0 | 26.4 | 2.7 | 18.0 | 26.4 | 2.7 | 18.0 | 31.8 | 5.4 | 36.0 | 9.0 | 4.5 | 30.0 | - | - | - |
|  | M | 132.0 | 60.0 | 120.0 | 26.4 | 2.7 | 18.0 | 26.4 | 2.7 | 18.0 | 26.4 | 2.7 | 18.0 | 31.8 | 5.4 | 36.0 | 9.0 | 4.5 | 30.0 | - | - | - |
| 2023 Spring | FP | 273.0 | 225.0 | 225.0 | 22.5 | 0.0 | 7.5 | - | - | - | - | - | - | 27.0 | 0.0 | 52.5 | - | - | - | - | - | - |
|  | OPT | 111.0 | 27.0 | 90.0 | 9.0 | 4.5 | 30.0 | 26.1 | 2.7 | 18.0 | 31.5 | 5.4 | 36.0 | 31.5 | 5.4 | 36.0 | 18.0 | 9.0 | 60.0 | 9.0 | 4.5 | 30.0 |
|  | PS | 111.0 | 42.0 | 90.0 | 9.0 | 4.5 | 30.0 | 26.1 | 2.7 | 18.0 | 31.5 | 5.4 | 36.0 | 31.5 | 5.4 | 36.0 | 18.0 | 9.0 | 60.0 | 9.0 | 4.5 | 30.0 |
|  | M | 111.0 | 56.6 | 90.0 | 9.0 | 4.5 | 30.0 | 26.1 | 2.7 | 18.0 | 31.5 | 5.4 | 36.0 | 31.5 | 5.4 | 36.0 | 18.0 | 9.0 | 60.0 | 9.0 | 4.5 | 30.0 |

Note: Not significant or zero input.

**Table S2. Water input at different growth stages under each treatment in autumn 2022 and spring 2023.**

| Year/Date | | Unit | 8/26 | 8/30 | 9/2 | 9/5 | 9/9 | 9/29 | 10/1 | 10/21 | 11/02 | 11/14 | - | - |
| --- | --- | --- | --- | --- | --- | --- | --- | --- | --- | --- | --- | --- | --- | --- |
| 2022 Autumn | FP | （m^3^ ha^-1^） | 169.1 | 117.5 | 116.5 | 79.2 | 79.2 | 87.5 | 50.0 | 50.0 | 100.0 | 75.0 | - | - |
|  | OPT | （m^3^ ha^-1^） | 169.1 | 117.5 | 116.5 | 79.2 | 79.2 | 53.2 | 47.9 | 71.8 | 75.6 | 52.2 | - | - |
|  | PS | （m^3^ ha^-1^） | 169.1 | 117.5 | 116.5 | 79.2 | 79.2 | 67.8 | 52.7 | 86.3 | 64.0 | 62.0 | - | - |
|  | M | （m^3^ ha^-1^） | 169.1 | 117.5 | 116.5 | 79.2 | 79.2 | 58.1 | 44.5 | 82.0 | 76.6 | 52.2 | - | - |
| Date | | Unit | 1/13 | 1/28 | 2/15 | 3/3 | 3/12 | 3/24 | 3/31 | 4/8 | 4/20 | 4/30 | 5/8 | 5/14 |
| 2023 Spring | FP | （m^3^ ha^-1^） | 264.7 | 126.0 | 151.2 | 113.4 | 112.5 | 99.8 | 187.5 | 142.5 | 187.5 | 187.5 | 150.0 | 112.5 |
|  | OPT | （m^3^ ha^-1^） | 264.7 | 126.0 | 151.2 | 113.4 | 67.3 | 76.5 | 114.4 | 122.0 | 147.0 | 158.7 | 134.3 | 111.3 |
|  | PS | （m^3^ ha^-1^） | 264.7 | 126.0 | 151.2 | 113.4 | 71.6 | 78.0 | 112.6 | 122.0 | 147.0 | 169.6 | 139.2 | 110.1 |
|  | M | （m^3^ ha^-1^） | 264.7 | 126.0 | 151.2 | 113.4 | 62.0 | 79.4 | 110.7 | 141.4 | 147.0 | 145.5 | 138.0 | 107.7 |

Note: Not significant or zero input.

# Supplementary Figures


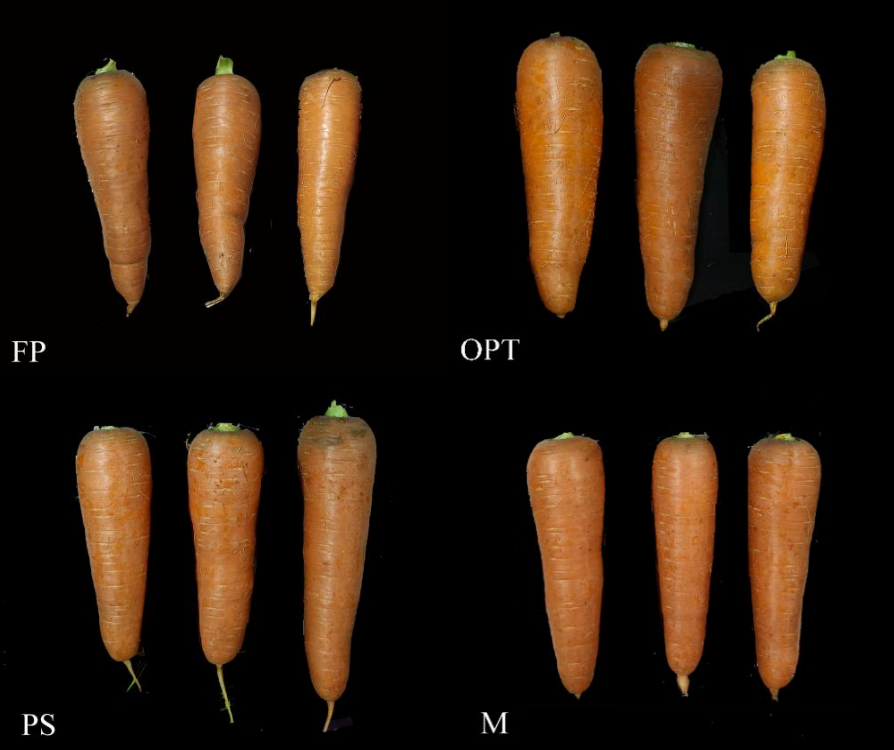


**Fig. S1. Comparison of the appearance of carrots under different treatments.**
